# Nitrogen form substitution identifies nitrogen use efficiency management pathways in maize

**DOI:** 10.1007/s12298-026-01753-z

**Published:** 2026-04-27

**Authors:** Joseph N. Amoah, Claudia Keitel, Brent N. Kaiser

**Affiliations:** School of Life and Environmental Sciences, The University of Sydney, 380 Werombi Road, Brownlow Hill, Camden, NSW 2570 Australia

**Keywords:** Diurnal nitrogen allocation, Maize growth and metabolism, Nitrate and ammonium dynamics, Nitrogen assimilation, Nitrogen use efficiency

## Abstract

Plants depend on nitrogen (N) for their growth, development, and metabolic functions. However, the regulatory mechanisms modulating N assimilate allocation under varying N forms are unclear. This study examines N metabolism and spatial distribution in maize seedlings subjected to four N treatments (T1 to T4): T1, 1 mM NO_3_^−^ (sole NO_3_^−^); T2, substitution of 1 mM NO_3_^−^ with 1 mM NH_4_^+^ (N form substitution, NFS); T3, 1 mM NH_4_^+^ (sole NH_4_^+^); and T4, 0.5 mM NH_4_NO_3_ (mixed N supply). The NFS treatment induced significant physiological and molecular adaptations, such as enhanced growth and total biomass under fluctuating N conditions. NFS-treated plants exhibited improved photosynthesis, increased protein and amino acid synthesis, and increased NO₃⁻ and NH₄⁺ accumulation. Activities of key N metabolism enzymes, such as nitrate reductase (NR), nitrite reductase (NiR), glutamine synthetase (GS), and glutamate synthase (GOGAT), were significantly upregulated, supporting efficient assimilation of both NO_3_^−^ and NH_4_^+^. Furthermore, spatial and diurnal analyses revealed dynamic N partitioning and adaptive regulation, with NFS-treated plants maintaining consistently higher NO_3_^−^ and NH_4_^+^ levels in leaves, roots, sheaths, and developing ears. These findings highlight the robust plasticity of maize N metabolism under NFS conditions and provide valuable insights into optimizing N use efficiency (NUE) for sustainable crop production. Future studies will focus on exploring these adaptive mechanisms across different maize genotypes and under field conditions to improve NUE and productivity in varying N environments.

**Supplementary Information:**

The online version contains supplementary material available at 10.1007/s12298-026-01753-z.

## Introduction

Nitrogen (N) is an important macro-nutrient nutrient required for the growth, development, and yield of crops. In agricultural soils, nitrate (NO_3_^−^) and ammonium (NH_4_^+^) are the two primary forms of N available to plants, with NO_3_^−^ typically being the predominant source due to its higher concentration (George et al. 2016). The positive impact of NO_3_^−^ on plant growth and metabolism has been widely documented across various species. For instance, NO_3_^−^ treatment has been associated with enhanced growth (Yan et al. 2023), increased N accumulation, and improved N use efficiency (NUE) (Peng et al. 2023b; Amoah et al. 2025c). It also leads to higher total amino acid and protein content (Liang et al. 2023), contributing to better quality, increased harvestable yields (Wei et al. 2024) and improved sugar metabolism (Peng et al. 2023a; Amoah et al. 2025b). The activity of nitrate reductase (NR), a crucial enzyme for NO_3_^−^ assimilation, is also tightly regulated, further optimizing N assimilation in plants (Fu et al. 2023; Wang et al. 2024).

NH_4_^+^ nutrition has been a nutrition form associated with mixed effects on plant growth and metabolism. While it can positively influence certain physiological and biochemical processes, its adverse impacts often outweigh the benefits in many crop species, although these effects are highly dependent on various factors including plant species, growth stage, soil pH, nutrient concentration, and environmental conditions. Documented positive effects of NH_4_^+^ include increased growth, improved N assimilation, and enhanced NUE in maize (George et al. 2016), improved salinity tolerance in sorghum (de Souza Miranda et al. 2016), improved grain quality in wheat (Fuertes-Mendizábal et al. 2013), enhanced photosynthesis in rice (Guo et al. 2007), as well as enhanced N metabolism and amino acid synthesis in tomato (González-Hernández et al. 2019). However, excessive NH_4_^+^ supply can negatively affect plant performance due to its toxicity, leading to metabolic imbalances and increased energy demand for detoxification (Hachiya et al. 2021). Studies have shown that NH_4_^+^ reduces growth and NO₃⁻ accumulation, reduces N assimilation, inhibits photosynthesis and reduces yield quality (Wang et al. 2019a; Chen et al. 2023; Peng et al. 2023b; Amoah et al. 2025a).

Although the comparative effects of NH_4_^+^ and NO_3_^−^ on various metabolic processes are well-documented, studies show that combining these N forms in varying ratios enhances plant growth and improves N uptake and utilization more effectively than supplying either form alone. This enhanced growth is attributed to the synergistic interaction between NO_3_^−^ and NH_4_^+^ (Wang et al. 2019b; Peng et al. 2023a; Chen et al. 2024a). While plants generally prefer NH_4_^+^ due to its lower energy requirement for assimilation (Hachiya and Sakakibara 2017), little is known about the impacts of dynamically switching between these nutrient forms. In natural and agricultural settings, NO_3_^−^ and NH_4_^+^ availability fluctuates due to microbial activity, fertilization practices, and environmental changes (Norton and Ouyang 2019; Hui et al. 2024). Prolonged fluctuations can trigger stress responses that may impair overall productivity. However, static studies fail to fully capture these dynamic conditions. Previous research on plant N management has primarily focused on static N supply or single N forms (Garnett et al. 2013; George et al. 2016; Plett et al. 2016; Dechorgnat et al. 2019; Wang et al. 2019b; Peng et al. 2023a). This approach overlooked the complexity of changing N availability, leaving a gap in understanding plant responses to dynamic N conditions.

Nitrogen Form Substitution (NFS) treatments tackle this limitation by integrating alternating N sources rather than maintaining a constant N environment. By simulating natural N fluctuations, NFS provides new perspective into how plants adapt to varying N conditions and optimize their growth. Unlike static studies, which capture only responses to unchanging N supply, NFS offers a more comprehensive understanding of nutrient adaptation mechanisms. In our recent study, NFS treatments significantly promoted growth, enhanced photosynthesis, and stimulated carbon metabolism in maize. The observed growth responses and metabolic adjustments highlight adaptive strategies and synergistic interactions that were not evident in plants exposed to a single N form (Amoah and Kaiser 2025b). These findings underscore the importance of dynamic nitrogen availability in shaping plant performance and reinforce the value of NFS as an approach for investigating nutrient adaptation mechanisms. Building on these insights, the present study was designed to explore how NFS influences N metabolism in maize. By integrating changes in both N form and supply, we aimed to elucidate the regulatory mechanisms modulating N uptake, assimilation, and utilization under fluctuating N conditions, thereby advancing our understanding of plant adaptive responses to dynamic N environments and provide valuable insights for breeding crops with improved nitrogen use efficiency (NUE).

## Materials and methods

### Plant materials and experimental site

Seeds of the fast-flowering, short-cycle inbred mini-maize line TX-40 J (McCaw et al. 2016), which were used in our previous experiment (Amoah et al. 2025a), were also used in this study. Seeds were disinfected with 5% sodium hypochlorite for 5 min and washed with ultrapure water, 5 times at 3 min each. Sterilized maize seeds were germinated in Oasis Horticube Propagation Slabs (Aqua Gardening, Australia), an inorganic and pH-neutral growing foam medium, placed in germination trays.

### Experimental treatment, set up and sampling

**Fig. 1:**
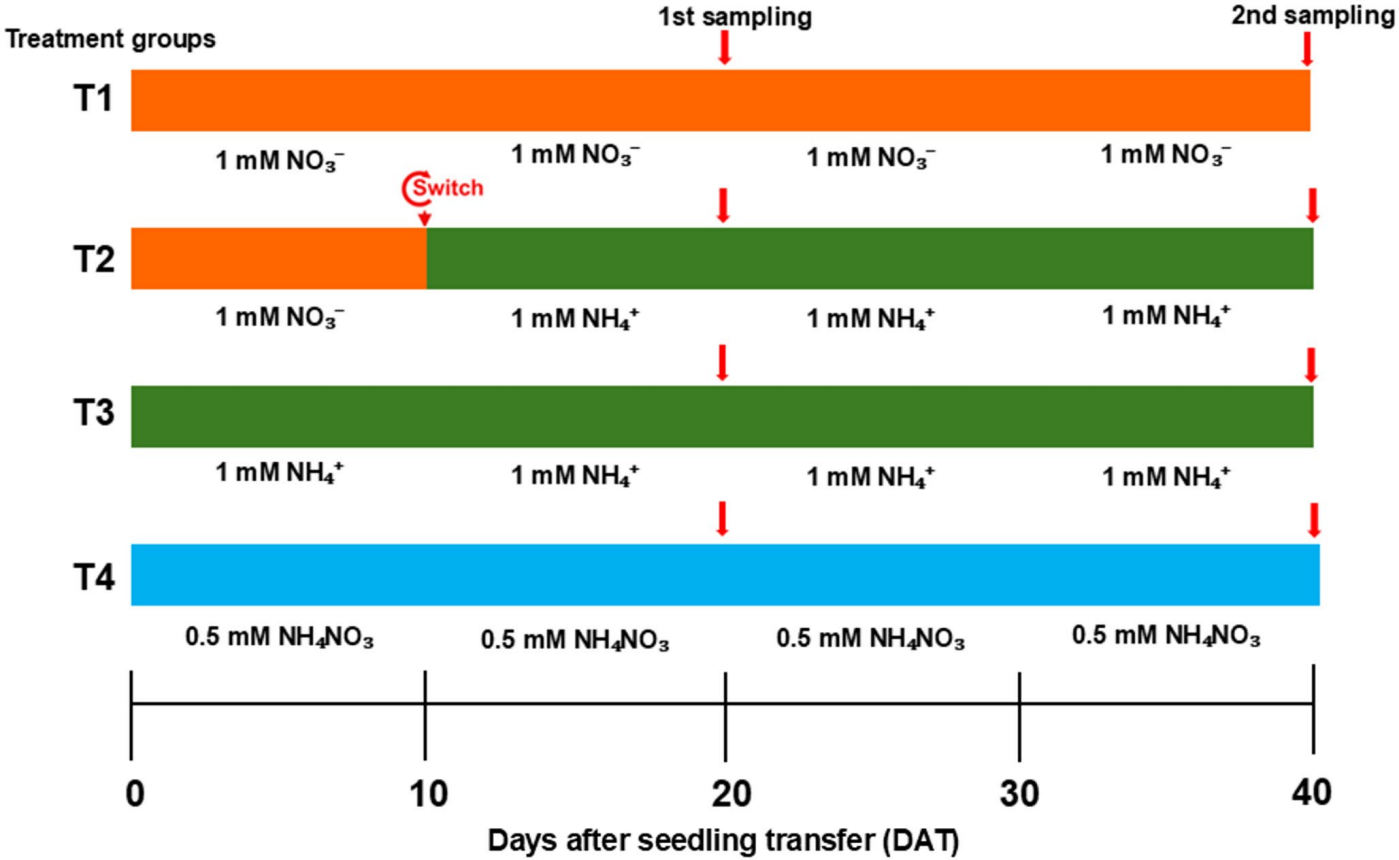
Schematic representation of the experimental setup for the maize inbred line TX-40 J. Maize seedlings were assigned to four nitrogen (N) treatment groups (T1 to T4): T1, 1 mM NO_3_^−^ (sole nitrate supply); T2, substitution of 1 mM NO_3_^−^ with 1 mM NH_4_^+^ at 10 days after transfer (DAT) (nitrogen form substitution, NFS); T3, 1 mM NH_4_^+^ (sole ammonium supply); and T4, 0.5 mM NH_4_NO_3_ (combined nitrate and ammonium supply). Plants were subsequently grown for an additional 30 days (up to 40 DAT), with shoot, root, and leaf samples collected at 20 DAT and 40 DAT. In the diagram, orange, green, and blue blocks represent 1 mM NO_3_^−^, 1 mM NH_4_^+^, and 0.5 mM NH_4_NO_3_, respectively. Red downward arrows indicate the nitrogen form switch (from 1 mM NO_3_^−^ to 1 mM NH_4_^+^), while black downward arrows denote tissue sampling time points

Seedlings were categorized into four treatment groups (T1–T4) and cultivated in 3 L pots, with their roots stabilized using inorganic expanded clay pellets (Aquaponics, Perth, Australia). At the fully expanded third leaf stage (Fig. 1), plants in T1 and T2 were initially provided with 1 mM NO_3_^−^, whereas those in T3 and T4 received 1 mM NH_4_^+^ and 0.5 mM NH_4_NO_3_, respectively. While T1, T3, and T4 continued under the same nutrient conditions, T2 underwent the nitrogen form substitution (NFS) treatment, in which 1 mM NO_3_^−^ was substituted with 1 mM NH_4_^+^ (Amoah & Kaiser, 2025). Consequently, T2 served as the primary group for evaluating NFS effects on N uptake, assimilation, and utilization in maize, whereas T1, T3, and T4 functioned as reference groups for comparative analysis, aligned with prior investigations (Amoah and Kaiser 2025b). Low concentrations of NO_3_^−^, NH_4_^+^, and NH_4_NO_3_ were employed to better reflect field conditions, mitigate NH_4_^+^ toxicity, and enable a more precise evaluation of N assimilation. This approach facilitates the assessment of NUE, highlights key physiological and molecular adaptations, and allows for a detailed analysis of NO_3_^−^ and NH_4_^+^ interactions (George et al. 2016; Ye et al. 2022). Plants were cultivated for 40 d after seedling transfer (DAT), with tissue sampling conducted at 20 (vegetative: V6 stage) and 40 (reproductive: R1 stage) DAT (Amoah and Kaiser 2025a). The system was established in a climate-controlled glasshouse under environmental conditions identical to those of the seed germination growth room, with supplemental LED lighting delivering 1000 µmol m^−2^ s^−1^ at pot level. Each system accommodated 40 pots, with one plant per pot. Nutrient solutions were delivered via a drip-irrigation system integrated with a hydroponic pump to ensure circulation. Irrigation was administered twice daily for 1 min, at 12:00 and 17:00 (Amoah and Kaiser 2025b).

The nutrient solution contained the following concentrations (mM): 1.0 KNO_3_, 1.0 (NH_4_)_2_SO_4_, 0.5 NH_4_NO_3_, 1.0 MgSO_4_, 1.0 KH_2_PO_4_, 0.05 H_3_BO_3_, 0.005 MnSO_4_, 0.001 ZnSO_4_, 0.001 CuSO_4_, 0.001 Na_2_MoO_4_, 0.1 KCl, 0.1 Fe-EDTA, 0.1 Fe-EDDHA, 0.25 Ca(NO_3_)_2_, 0.25 K_2_SO_4_, 0.25 CaCl_2_, and 1.75 CaSO_4_ (Amoah and Kaiser 2025a). In treatments with reduced NO_3_^−^ supply, the decrease in Ca(NO_3_)_2_ was compensated by equivalent additions of CaCl_2_ and CaSO_4_ to maintain consistent Ca^2+^ availability across treatments. The solution was stored in 162 L Brute Containers with lids (Rubbermaid, USA) and replaced weekly, with daily pH adjustments using 1 M H_2_SO_4_ or 1 M NaOH to maintain a stable pH of 5.9. The treatment solution was delivered via an Eden 140G FL submersible water pump (Creative Pumps, Australia). A randomised complete block design (RCBD) was used in this study, with six (6) biological replicates per treatment. Each pot contained a single plant and was randomly assigned to blocks, using the ‘*agricolae*’ package of the R statistical software (v4.4.5) to minimize positional bias. Plants were uniquely identified and randomized into blocks using R statistical software (v4.4.5). Fresh leaf, root, and ear tissues designated for biochemical analysis were immediately frozen in liquid nitrogen (N_2_) and stored at − 80 °C. Shoot and root samples for biomass analysis were oven-dried at 70 °C for 48 h to determine biomass accumulation (DW). Total plant biomass (g) was calculated by summing shoot and root biomass values.

### Determination of spatial distribution and diurnal variations

To investigate the spatial distribution of NO_3_^−^ and NH_4_^+^, as well as NR and GS activities, the youngest emerging leaf was sampled at 20 and 40 DAT and segmented into upper and middle sections. Additional samples were taken from the corresponding leaf sheath, root, and developing ear. At 40 DAT, sampling was conducted at 22:00 and again at 7:00 the following morning. For diel analysis, the middle sections of the youngest fully expanded leaves were collected at five time points, at 7:00, 12:00, 17:00, 22:00, and again at 7:00 the next day, at both 20 and 40 DAT. All samples were immediately frozen in liquid nitrogen (N_2_) and stored at − 80 °C for subsequent biochemical analysis.

### Photosynthetic rate, chlorophyll, and nitrogen determination

Photosynthetic rate (Pn ) was measured on the youngest emerging leaf of each treatment using a LI-6400 portable photosynthetic system (LI-COR Inc., Lincoln, NE, USA). Measurements were recorded at 10:00 AM and 12:00 PM under cuvette conditions of 1000 µmol m^−2^ s^−1^ light intensity, 400 ppm CO_2_ concentration, a flow rate of 500 µmol m^−2^ s^−1^, and RH maintained between 60% and 65%. For chlorophyll content pigment extraction, 100 mg of leaf tissue was mixed with 100% methanol and centrifuged at 5,000 × g for 5 min. The absorbance of the supernatant was measured at 646, 470, and 663 nm using a spectrophotometer (Shimadzu, Japan). N content was quantified using the Kjeldahl method (Amoah & Kaiser, 2025), with slight modifications. A 0.1 g dry sample was digested with 0.5 mL concentrated H_2_SO_4_ and 0.5 mL of a catalyst mix containing 10 g K_2_SO_4_ and 1 g CuSO_4_ and heated at 90 °C for 1 h. The mixture was cooled, and 0.5 mL of 40% NaOH was gradually added, followed by 500 µL of distilled water. Next, 1 mL of the solution was added to 1 mL of Nessler’s reagent and incubated for 10 min at room temperature. Absorbance was measured at 420 nm using a UV-Vis spectrophotometer (Shimadzu, Japan), and N content was determined using a standard curve generated from (NH_4_)_2_SO_4_ standards.

### Total amino acid and protein quantification

Tissue amino acid content was assayed following the method outlined by Amoah and Kaiser (2025b). Frozen leaf tissues (100 mg) were homogenized in 10 mL of 3% (v/v) aqueous sulfosalicylic acid. After filtration, 1 mL of the filtrate was mixed with 1 mL of glacial acetic acid and 1 mL of acidic ninhydrin, then incubated at 100 °C for 1 h. The solution was cooled for 20 min, followed by the addition of 1 mL of toluene. Amino acid concentrations were measured at 580 nm using a spectrophotometer. Total protein content in leaf and root tissues was extracted from 100 mg of fresh material using the phenol approach outlined by Dechorgnat et al. (2018). After centrifugation, the supernatant was used for enzyme activity assays.

### Nitrate (NO_3_^−^), nitrite (NO_2_⁻) and ammonium (NH_4_⁺) determination

NO₃⁻, NO₂⁻, and NH₄⁺ were extracted from 100 mg of dried plant material using 1 mL of water. NO₃⁻ content was quantified using a colorimetric assay based on the reduction of NO_3_^−^ to NO_2_^−^ by Vanadium (III) (Dechorgnat et al. 2018). NO_2_^−^ levels were determined using the Griess assay (Dechorgnat et al. 2011), while NH₄⁺ concentrations were measured using the Ammonium Assay Kit (Sigma-Aldrich, St. Louis, MO, USA, cat# AA0100), following the manufacturer’s instructions.

### Nitrate reductase (NR), nitrite reductase (NiR), glutamine synthase (GS) and glutamate synthase (GOGAT) activities assay

NR activity was assayed using the method described by Dechorgnat et al. (2018), with minor modifications. A 10 µL aliquot of extracted protein solution was mixed with 90 µL of reaction buffer containing 130 mM K_2_HPO_4_, 70 mM KH_2_PO_4_, 0.5 mM KNO_2_, and 2 mM methyl viologen. The reaction was initiated by adding 10 µL of a sodium-based solution (20 mg/mL Na_2_CO_3_ and Na_2_S_2_O_4_) and incubated at 30 °C for 20 min. The reaction was terminated by vortexing, and NO_2_^−^ content was quantified using the Griess assay (Dechorgnat et al. 2018). GS and GOGAT activities were determined by mixing 20 µL of extracted protein with 80 µL of reaction buffer containing 100 mM hydroxylamine, 125 mM MOPS, 0.5 mM ADP, 12.5 mM sodium arsenate, 37.5 mM glutamine, and 1.25 mM MnCl_2_. The reaction was incubated at room temperature for 30 min, followed by the addition of 100 µL of detection buffer comprising 370 mM FeCl_3_, 576 mM HCl, and 157 mM TCA. Optical density was measured at 540 nm, with L-glutamic acid γ-monohydroxymate (GHA) serving as the reference standard (Dechorgnat et al. 2018). NiR activity was assessed by combining 10 mL of extracted protein solution with 90 mL of reaction buffer (130 mM K_2_HPO_4_, 70 mM KH_2_PO_4_, 0.5 mM KNO_2_, and 2 mM methyl viologen), followed by the addition of 10 mL of a sodium-based reaction solution containing 20 mg/mL Na_2_CO_3_ and Na_2_S_2_O_4_. After incubation at 30 °C for 15 min, the reaction was stopped by vertexing, and nitrite concentration was quantified using the Griess assay (Wang et al. 2007).

### Statistical analysis

The study was independently conducted twice, with tissue samples collected in triplicate for each experiment. All quantitative data represent the mean ± deviation (SD) of six (6) independent plants (*n* = 6). Data were analysed using a two-way analysis of variance (ANOVA) to assess the effects of treatment and developmental stage. Post-hoc pairwise comparisons were subsequently performed in GraphPad Prism (v10.4.0) to identify statistically significant differences between groups. In the ANOVA model, plant growth stage (PGS) was designated as a fixed factor, while N treatments (NT) were treated as variable factors. Statistical significance was determined at a probability of *P* ≤ 0.05, with differing lowercase letters used to denote significant differences in graphical representations. Graphical visualizations were generated using GraphPad Prism (v10.4.0).

## Results

### Phenotypic performance and biomass accumulation under dynamic N forms

**Fig. 2:**
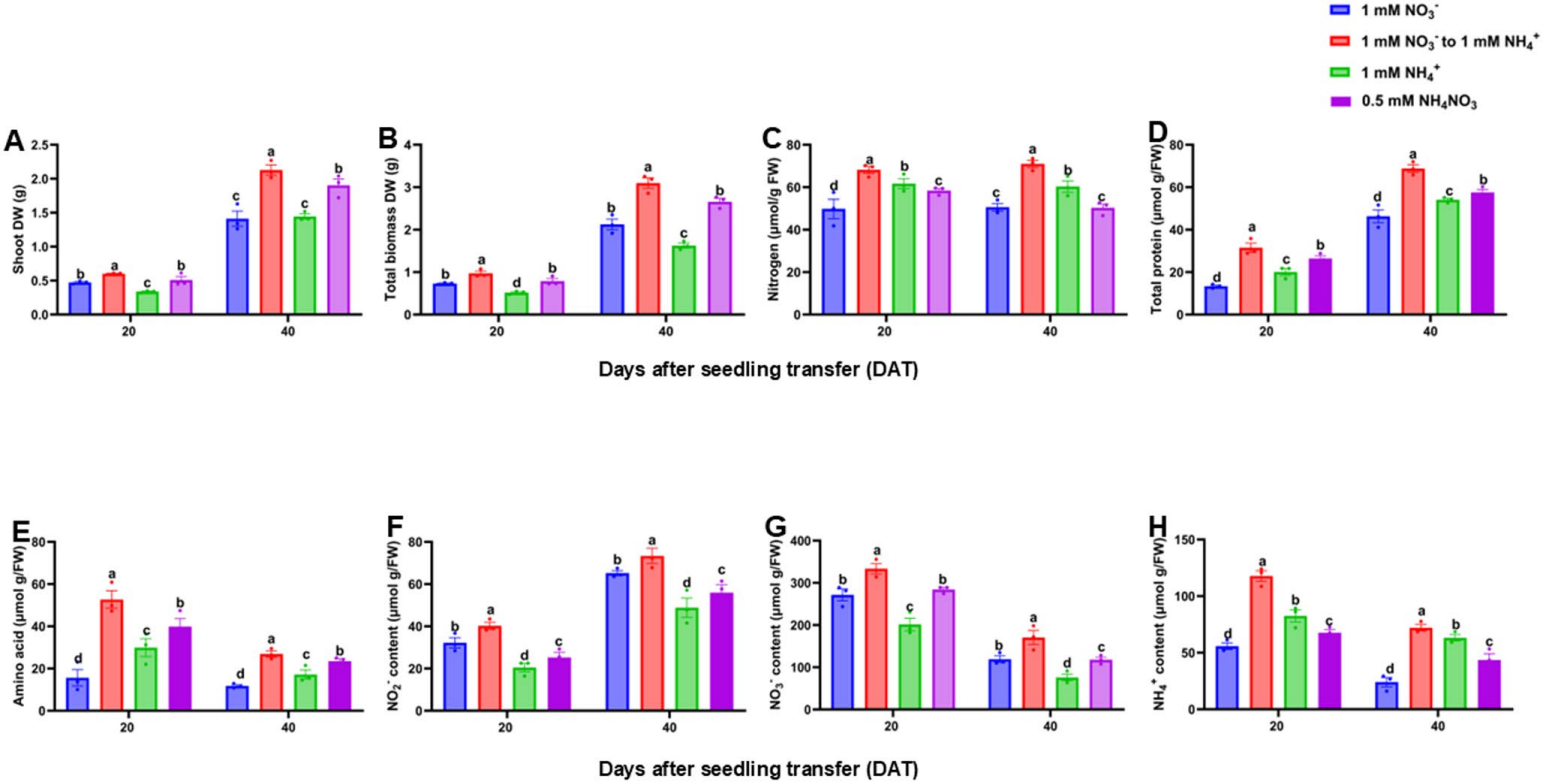
Shoot biomass (**A**), total biomass (**B**), leaf nitrogen content (**C**), leaf total protein content (**D**), leaf total amino acids (**E**), leaf nitrate (**F**), leaf nitrite (**G**), and leaf ammonium content (**H**) under different nitrogen forms. Data points represent the mean ± standard deviation (SD) of six independent biological replicates (*n* = 6). Different letters above the error bars indicate statistically significant differences at *P* ≤ 0.05. *DAT * days after seedling transfer;* FW* fresh weight;* DW* dry weight

Maize seedlings showed distinct growth responses to the various N treatments. By 20 DAT, plants subjected to the NFS treatment displayed significantly improved shoot and root growth, resulting in superior overall phenotypic performance compared to those grown under other N treatments (Fig. S1A and B). This enhanced growth trend continued at 40 DAT, with NFS-treated plants displaying vigorous shoot development and produced larger cobs. Conversely, plants grown solely with NH_4_^+^ developed more extensive root systems than those under other N regimes (Fig. S2A and B). While this highlights the differential effects of N sources on maize growth dynamics, the R/S ratio remained stable across all treatments and growth stages (sampling points). This suggests that the visually larger roots observed in plants grown under NH_4_^+^ treatment did not result in a statistically significant change in the proportion of root to shoot biomass. Furthermore, NFS treatment significantly enhanced total N accumulation in both leaf and root tissues at 20 and 40 DAT (Fig. 2 C). This increase was accompanied by a substantial rise in shoot, root, and total biomass compared to plants supplied with sole NO_3_^−^, NH_4_^+^, or mixed (NH_4_NO_3_) N sources. Despite this significant absolute increase in biomass accumulation, the R/S ratio remained stable across all treatments and time points (Fig. 2 A, B, and D), suggesting proportionally coordinated growth. In parallel, NFS-treated plants exhibited elevated chlorophyll content and higher Pn relative to other N treatment (Fig. S3A and B). Two-way ANOVA revealed that both plant growth stage (PGS) and nitrogen treatment (NT) significantly influenced total biomass and individual components of biomass partitioning (root and shoot), N content, chlorophyll concentration, and Pn. Moreover, a significant PGS × NT interaction was observed for total biomass, root biomass, as reflected in significant effect on absolute shoot, root, and total biomass, as well as on and root N content. However, the R/S ratio remained unaffected by either factor or their interaction according to the two-way ANOVA (Table 1). This demonstrates that while NFS promotes significantly greater absolute biomass production in both shoot and roots, the relative proportion of mass allocated between these tissues is proportionally maintained.

**Fig. 3:**
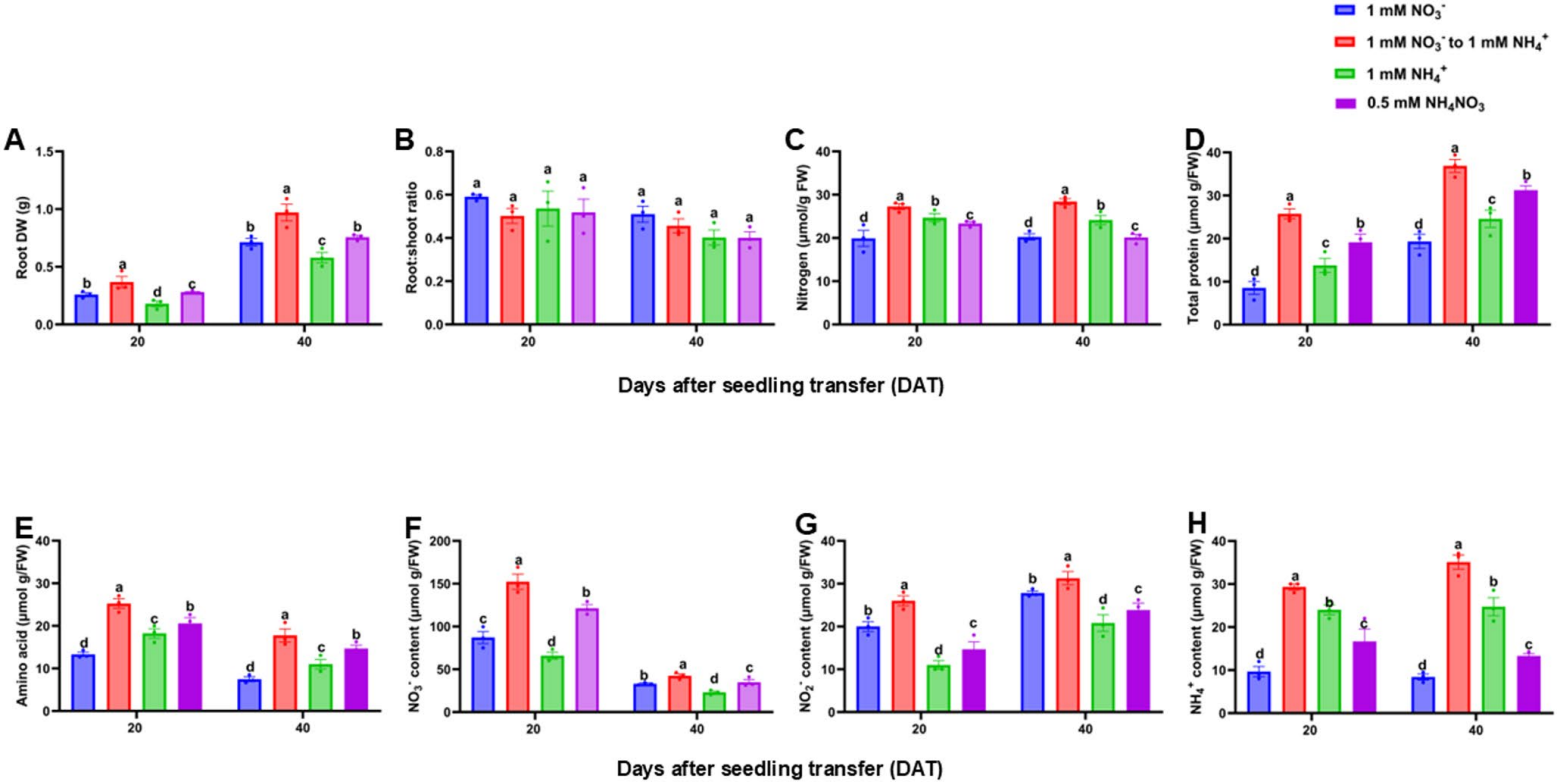
Root biomass (**A**), root: shoot biomass ratio (**B**), root nitrogen content (**C**), root total protein content (**D**), root total amino acids (**E**), root nitrate (**F**), root nitrite (**G**), and root ammonium content (**H**) under different nitrogen forms. Data points represent the mean ± standard deviation (SD) of six independent biological replicates (*n* = 6). Different letters above the error bars indicate statistically significant differences at *P* ≤ 0.05. *DAT* days after seedling transfer;* FW* fresh weight;* DW* dry weight

**Table 1:** Two-way analysis of variance (ANOVA) for the indicators in the shoot/leaf and root of maize inbred-line TX-40J under different N treatments

Trait/(plants)	Sources of variation					
	Shoot/leaf			Root		
	PGS (df=1)	NT (df=3)	PGS x NT (df=3)	PGS (df=1)	NT (df=3)	PGS x NT (df=3)
Biomass	0.0004***	0.0462*	0.0651ns	0.0075**	0.0051**	0.0847ns
Total biomass	0.0005***	0.0275*	0.0367*	0.0005***	0.0275*	0.0367*
Root: shoot ratio	0.1615ns	0.3714ns	0.8599ns	0.1615ns	0.3714ns	0.8599ns
Chlorophyll	0.017*	0.0454*	0.2045ns	n/a	n/a	n/a
Pn	0.02921*	0.0487*	0.2679ns	n/a	n/a	n/a
Nitrogen	0.02474*	0.0286*	0.3226ns	0.03474ns	0.0286*	0.2226ns
Total protein	0.0003***	0.0094**	0.0898ns	0.01293*	< 0.0001****	< 0.0001****
Total amino acid	0.0034**	< 0.0001****	< 0.0001****	0.0099**	< 0.0001***	< 0.0001*****
Nitrate	0.0318*	< 0.0001****	< 0.0001****	< 0.0001****	< 0.0001****	< 0.0001****
Nitrite	0.02739ns	< 0.0001****	< 0.0001****	0.0106*	< 0.0001****	0.0001***
Ammonium	0.0004***	< 0.0001****	< 0.0001****	0.0015**	0.0073**	< 0.0001****
NR	0.0294*	< 0.0001****	< 0.0001****	0.0116*	0.0004***	< 0.0001***
GS	0.0003***	< 0.0001****	< 0.0001****	0.001***	0.0867ns	< 0.0001****

PGS, plant growth stage; NT, nitrogen treatment; PGS x NT, interaction between plant growth stage and nitrogen treatments; Pn, net photosynthetic rate; NR, nitrate reductase activity; GS, glutamine synthetase activity; ns, non-significant; n/a, non-applicable. Additionally, *, **, and ***: denote significance at probability levels of 0.05, 0.01, and 0.001, respectively.

### NFS enhances amino acid and protein synthesis and N metabolite accumulation

The dynamic NFS treatment significantly increased the total amino acid and protein contents in both leaves and roots of maize seedlings at 20 and 40 DAT (Figs. 2, 3D and E). Similarly, NFS-treated plants accumulated higher levels of NO_3_^−^, NO_2_^−^ and NH_4_^+^ in both leaves and roots at both time points compared to all other N treatments (Fig. 2 F and H). The accumulation of these N metabolites in leaf and root tissues was significantly influenced by PGS, NT, and PGS × NT.

### Promotion of N assimilation enzyme activities by NFS

**Fig. 4:**
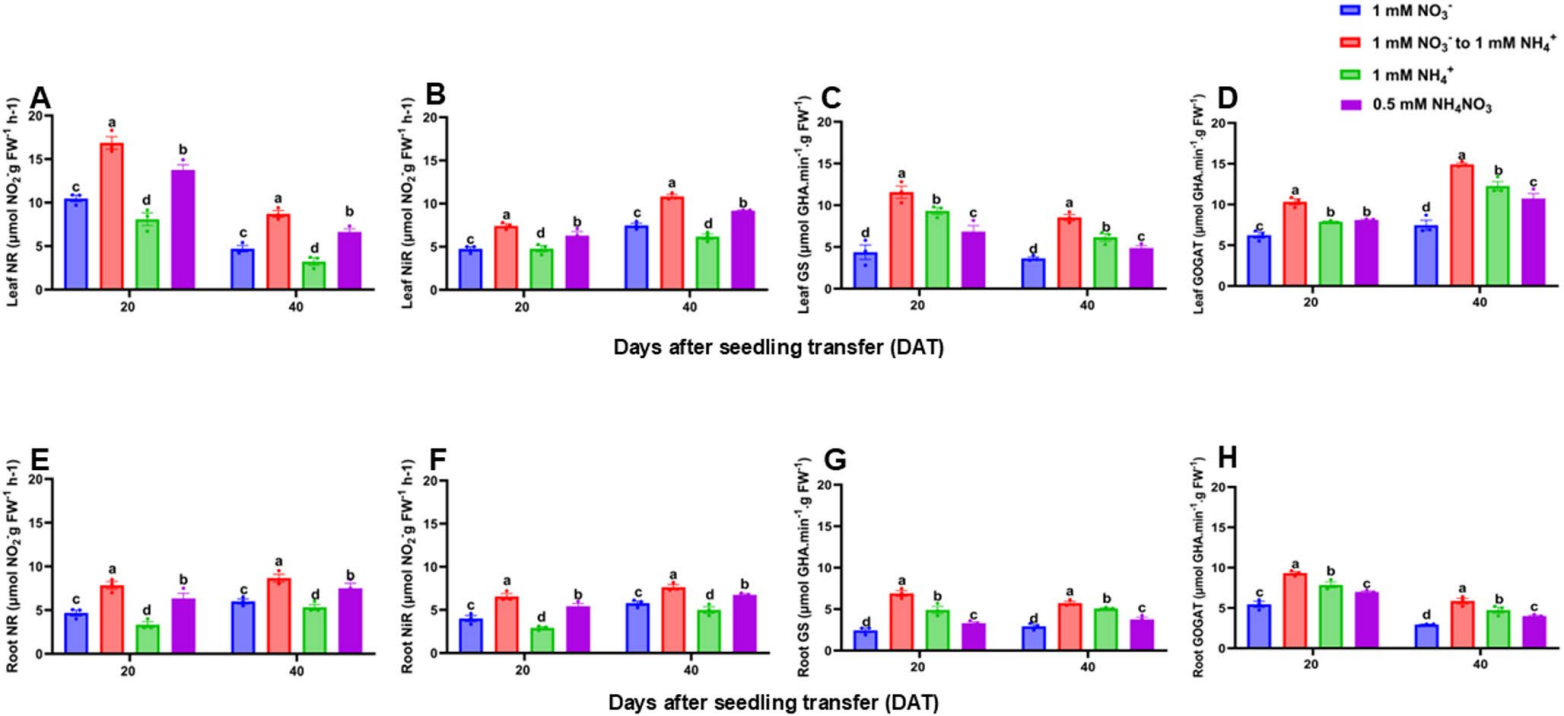
Nitrate reductase (**A**,** E**), nitrite reductase (**B**,** F**), glutamine synthetase (**C**,** G**), and glutamate synthase (**D**,** H**) activities in the leaves (**A**–**D**) and roots (**E**–**H**) of the maize inbred line TX-40 J. Data are presented as mean ± standard deviation (SD) from six independent biological replicates (*n* = 6). Different letters above the error bars indicate statistically significant differences at *P* ≤ 0.05. *DAT* days after seedling transfer;* FW* fresh weight

Consistent with the increased accumulation of N metabolites, the activities of key N assimilation enzymes, such as NR, NiR, GS and GOGAT, were significantly higher in the leaves and roots of NFS-treated plants compared to other N treatments (Fig. 4A–H). In leaves, the activities of NR, NiR, GS, and GOGAT were all significantly influenced by PGS, NT, and PGS × NT (Table 1). Additionally, in roots, NR, NiR, and GS activities were similarly affected by PGS, NT, and PGS × NT, while GOGAT activity was influenced solely by PGS and NT, with no significant interaction effect (Table 1).

### Diurnal patterns of N metabolites and enzyme activities

**Fig. 5:**
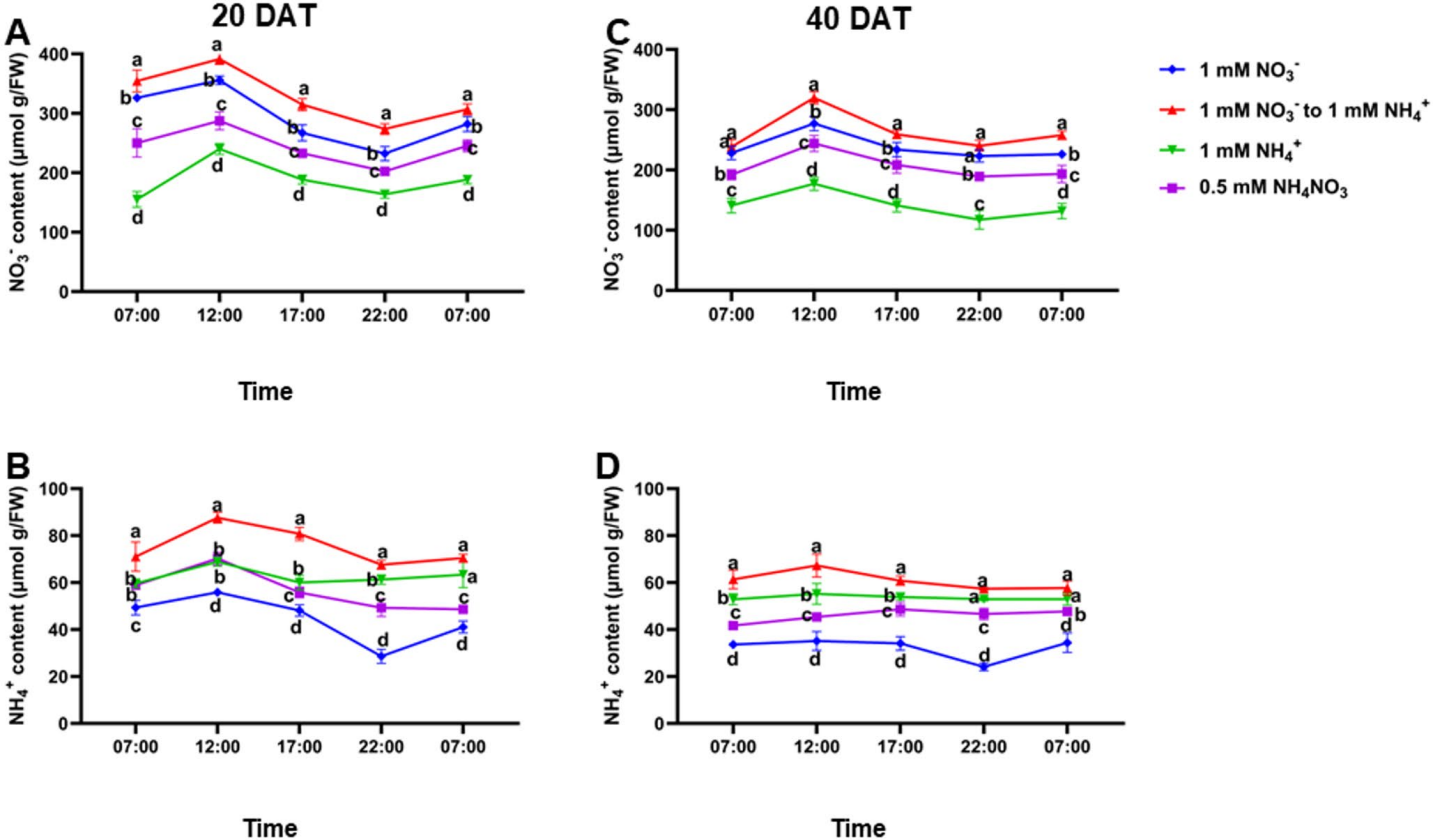
Diurnal changes in leaf nitrate and ammonium content under nitrogen form substitution (NFS) conditions. Leaf nitrate content at 20 days after transfer (DAT) (**A**) and 40 DAT (**B**), and leaf ammonium content at 20 DAT (**C**) and 40 DAT (**D**). Samples were collected at five time points: 7:00, 12:00, 17:00, 22:00, and 7:00 on the following day. Data are presented as mean ± standard deviation (SD) from six independent biological replicates (*n* = 6). Different letters above the error bars indicate statistically significant differences at *P* ≤ 0.05.* DAT* days after seedling transfer;* FW* fresh weight

**Fig. 6:**
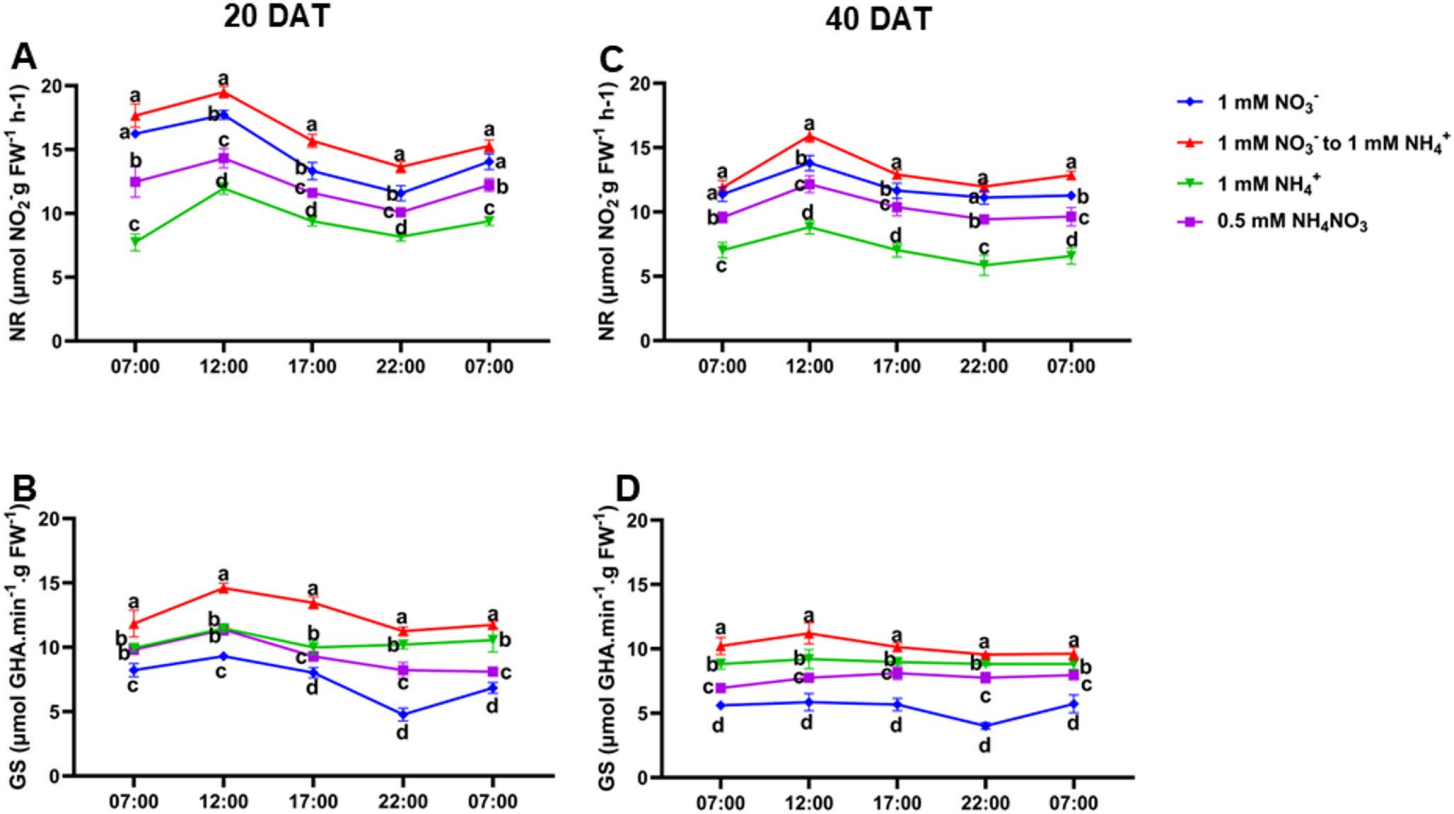
Diurnal changes in leaf nitrate reductase and glutamine synthase activity under nitrogen form substitution (NFS) conditions. Leaf nitrate reductase activity at 20 days after transfer (DAT) (**A**) and 40 DAT (**B**), and leaf glutamine synthase activity at 20 DAT (**C**) and 40 DAT (**D**). Samples were collected at five time points: 7:00, 12:00, 17:00, 22:00, and 7:00 on the following day. Data are presented as mean ± standard deviation (SD) from six independent biological replicates (*n* = 6). Different letters above the error bars indicate statistically significant differences at *P* ≤ 0.05. *DAT* days after seedling transfer;* FW* fresh weight

We observed consistent diurnal patterns of NO_3_^−^ and NH_4_^+^ accumulation and enzyme activities across treatments. Diurnal NO₃⁻ levels increased in the early morning (7:00 AM), peaked at midday (12:00 PM), and then declined until the following morning (Fig. 5A and B). Conversely, NH₄⁺ levels were lowest at 7:00 AM, increased after 12:00 PM, peaked at 10:00 PM, and then dropped by 7:00 AM the next day (Figs. 5B–D). These diurnal fluctuations were consistent at both 20 and 40 DAT (Figs. 5A–D). At 20 DAT, NFS-treated plants consistently exhibited significantly higher (*P* ≤ 0.05) accumulation of both NO_3_^−^ and NH_4_^+^ throughout the day compared to other treatment groups. Even after overnight transport, NFS-treated plants retained higher NO₃⁻ and NH₄⁺ levels (Fig. 5). Correspondingly, NR activity was high at 7:00 AM, peaked at 12:00 PM, and then rapidly decreased until the next morning (Fig. 6A–C). GS activity followed a similar pattern, increasing after 7:00 AM, peaking at 12:00 PM, and declining afterwards. At 20 DAT, NFS-treated plants consistently showed significantly higher (*P* ≤ 0.05) NR activity throughout the day compared to other treatments (Fig. 6).

### Spatial distribution of N forms across maize tissues

**Fig. 7:**
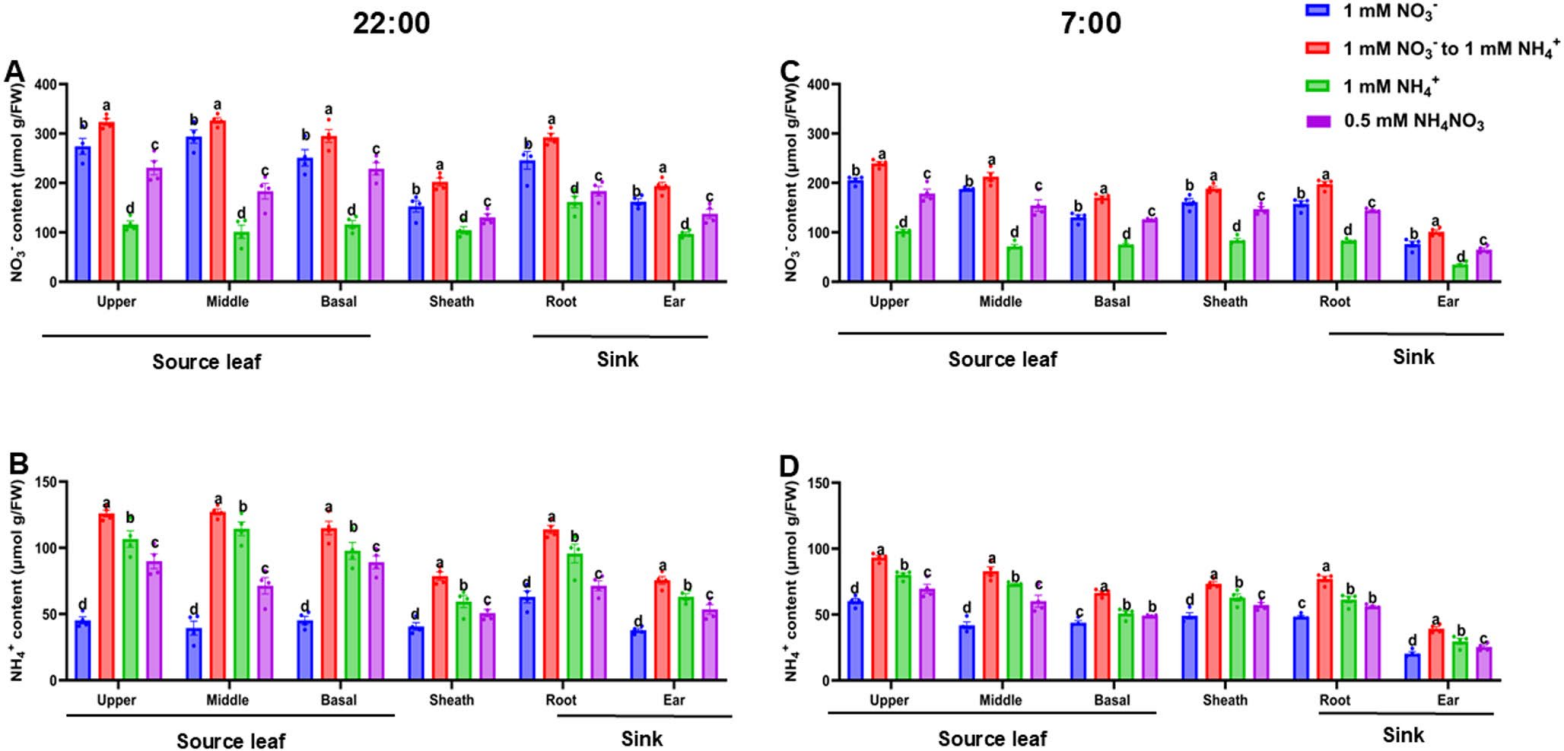
Spatial and temporal analysis of nitrate and ammonium content in maize tissues at 40 DAT. Leaf nitrate (**A**) and ammonium (**B**) levels at 22:00, and leaf nitrate (**C**) and ammonium (**D**) levels at 07:00, measured under different nitrogen form treatments. FW indicates the fresh weight of tissue samples. Data are presented as mean ± standard deviation (SD) from six independent biological replicates (*n* = 6). Different letters above the error bars denote statistically significant differences at *P* ≤ 0.05.* DAT* days after seedling transfer;* FW* fresh weight

Spatial analysis revealed differential distribution of NO_3_^−^ and NH_4_^+^ across various maize tissues, such as upper, middle, and basal leaves, leaf sheath, roots, and developing ear. NFS-treated plants exhibited significantly (*P* ≤ 0.05) higher concentrations of both NO_3_^−^ and NH_4_^+^ across these tissues compared to other N treatments. At 22:00, elevated NO_3_^−^and NH_4_^+^ levels were observed in the upper, middle, and basal leaves, along with the roots, suggesting enhanced N uptake and retention under NFS. Notably, NFS-treated plants maintained elevated NH_4_^+^ concentrations following overnight remobilization and increased NO_3_^−^ accumulation in leaves by 22:00. In sink tissue, such as the roots, NO_3_^−^ and NH_4_^+^ levels were comparatively higher and showed significant variation (*P* ≤ 0.05) among N treatments at both 22:00 and 07:00 (Fig. 7A-D), suggesting differential N dynamics throughout the diurnal cycle.

## Discussion

### NFS promotes maize growth by optimizing N allocation coordinating C partitioning

Our findings demonstrate that NFS significantly promotes shoot, root, and total plant biomass accumulation in maize compared to static single or mixed N forms (Figs. 2A and B and 3A). This enhanced growth response aligns with our recent study which showed that NFS treatment promoted growth, enhanced photosynthesis and stimulated C metabolism in maize (Amoah and Kaiser 2025b). While previous studies have shown that mixed NO_3_^−^ and NH_4_^+^ supply generally improves N uptake and utilization through synergistic interactions, our study provides an insight into how the dynamic and alternating nature of NFS specifically modulates this synergy to optimize plant performance and nutrient homeostasis under fluctuating conditions (Peng et al. 2023a; Chen et al. 2024b). The enhanced growth observed under NFS may be attributed to a complementary N uptake mechanism that mitigates stress and toxicity associated with sole N forms (Fang et al. 2021). The inclusion of NH_4_^+^, which requires less energy for uptake and assimilation than NO_3_^−^, contributes to optimized resource partitioning (George et al. 2016). For instance, NH_4_^+^ uptake can reduce the metabolic demand for NO_3_^−^ assimilation, allowing greater carbohydrate retention in shoots and promoting shoot biomass accumulation (Peng et al. 2023a). Although NFS increased absolute shoot, root and total plant biomass, the R/S ratio was unchanged across treatments and growth stages (Figs. 2A and B and 3B). This stability suggests that biomass partitioning between roots and shoots was proportionally maintained, despite NFS plants accumulating greater biomass (Fig. 2B). Such proportional biomass allocation is consistent with our previous studies (Bloom et al. 1985; ÅGREN and FRANKLIN 2003; Amoah and Kaiser 2025b), indicating that maize maintains a balanced biomass distribution between shoots and roots to optimize C assimilation and nutrient acquisition under favourable nutrient conditions.

Furthermore, NH_4_^+^ treatment is known to stimulate root proton extrusion, which lowers rhizosphere pH and can improve NO_3_^−^ availability, uptake and accumulation (Zhao et al. 2020; Amoah and Kaiser 2025b; Hachiya and Sakakibara 2017). This physiological adjustment provides a mechanistic basis for the observed synergistic uptake effect under NFS, leading to higher NO_3_^−^ accumulation in leaves and roots. The dynamic shift in N supply under NFS appears to play a crucial role in modulating N assimilation pathways. It is worth noting that the higher NO_3_^−^ levels observed in NFS plants after the switch do not result from the conversion of NH_4_^+^ into NO_3_^−^ (Figs. 2 and 3G). Instead, they reflect the mobilization and retention of NO_3_^−^ accumulated during the initial NO_3_^−^ nutrition phase. Plants can store substantial amounts of NO_3_^−^ in the vacuole, which may persist even after external NO_3_^−^ availability declines (Miller et al. 2007; Miller and Cramer 2005). Moreover, NH_4_^+^ supply likely reduced the reliance of NFS plants on NO_3_^−^ reduction for nitrogen assimilation because NH_4_^+^ assimilation is energetically more efficient, as reported in previous studies (Cánovas et al. 2007; Hachiya and Sakakibara 2017). This reduced metabolic demand may favour the retention of internal NO_3_^−^ rather than its depletion (Bloom et al. 1992; Miller et al. 2007). Additionally, NH_4_^+^-induced rhizosphere acidification may enhance NO_3_^−^ mobilization and uptake, contributing to the elevated NO_3_^−^ levels (Britto and Kronzucker 2002). Trace amounts of NO_3_^−^ present in other salts in the nutrient solution may also support continued low-level NO_3_^−^ uptake.

### NFS optimizes N assimilation through coordinated enzyme activity

To understand the metabolic basis of plant adaptation under NFS, we assessed how NFS influences N assimilation at the enzymatic levels. Our results demonstrate that NFS treatment increased NO_3_^−^ and NO_2_^−^ accumulation (Figs. 2 F and G, 3F and G), which has not been previously observed under alternating N forms. While enhanced accumulation of these assimilates is known under different N treatment forms, the dynamic switch in NFS likely stimulated NO_3_^−^ accumulation beyond its metabolic demand, facilitating greater storage capacity. This observation aligns with studies demonstrating that combining NO_3_^−^ and NH_4_^+^ enhances N uptake and efficiency, and our data demonstrate that the dynamic switch between N sources further amplified this synergistic effect (George et al. 2016; Peng et al. 2023a). Furthermore, NFS plants accumulated even higher NH_4_^+^ levels than plants supplied with sole NH_4_^+^ (Fig. 2 H). This increased NH₄⁺ pool mechanistically linked to enhanced amino acid biosynthesis and significantly enhanced GS and GOGAT activities (Figs. 4C and D, G and H). This rapid conversion of NH_4_^+^ into amino acids, triggered by the alternating N supply, serves as an adaptive strategy or detoxification mechanism to mitigate potential NH_4_^+^ toxicity, which is a common physiological challenge under sole NH_4_^+^ nutrition (Cánovas et al. 2007; Liu and von Wirén 2017). Collectively, these findings highlight that NFS enhances N assimilation capacity by coordinating NO_3_^−^ storage, NH_4_^+^ detoxification, and the activities of key N‑assimilatory enzymes.

### Diurnal regulation of N metabolism under dynamic N availability

Although diurnal fluctuations in N pools and enzyme activities are well-documented in plant physiology, our study highlights how NFS distinctly modulates these rhythms and their underlying regulatory mechanisms beyond simple N status. NFS plants consistently maintained elevated NO_3_^−^ and NH_4_^+^ concentrations both during the day and overnight (Figs. 5A–D), suggesting that the dynamic N supply enhances the capacity for N storage and may modulate the amplitude of these fluctuations, potentially through circadian-regulated signalling or nutrient-sensing pathways (Macduff et al. 1997). The rhythmic patterns observed, such as NO₃⁻ peaking in the morning and declining by midday, and NR activity mirroring this trend, indicate a coordinated regulation of NO_3_^−^ reduction and accumulation that is maintained or even enhanced under NFS (Fig. 6). In contrast, the gradual increase of NH_4_^+^ levels throughout the day, peaking at night, and the corresponding increase in GS activity reaching maximum levels at night, suggest a circadian-driven regulatory mechanism that ensures sustained NH_4_^+^ assimilation into organic molecules, particularly when NO_3_^−^ availability may be lower (Tucker et al. 2004; Shanks et al. 2024). The ability of NFS plants to maintain altered N profiles, characterized by increased NO_3_^−^ and NH_4_^+^ levels even after overnight transport, underscores the plant’s robust adaptive responses to modified N sources (Amoah and Kaiser 2025b; Hasan et al. 2022). These findings provide insights into the intricate interplay between N availability, metabolic regulation, enzymatic activity, and diurnal rhythms, advancing our understanding of plant adaptation mechanisms under dynamic nutrient conditions.

### N availability shapes N assimilated dynamics across maize tissues: implications for allocation and remobilization

The spatial distribution of NO_3_^−^ and NH_4_^+^ provides insights into how maize adapts to varying N forms (Fang et al. 2007), particularly under dynamic NFS conditions. Our results demonstrate that NFS-treated plants exhibited significantly increased NO_3_^−^ and NH_4_^+^ levels across all sampled aboveground tissues (leaves and leaf sheath) and roots, particularly at 22:00 (Fig. 7). This highlights enhanced N accumulation and retention throughout plants under NFS. The significant variation in NO_3_^−^ and NH_4_^+^ levels in roots at different time points (22:00 and 07:00) demonstrates the active role of roots as N sinks during dynamic N changes. This suggests that under NFS, specific NRT and AMT transporters are likely upregulated to facilitate rapid N acquisition and storage (Dechorgnat et al. 2019; Duan et al. 2023). Furthermore, the consistent residual NO_3_^−^ in the leaves and sheath of NFS-treated plants between 22:00 and 07:00 indicates a strategy of NO₃⁻ retention as a temporary storage pool for subsequent remobilization during periods of metabolic demand. This tissue-specific N distribution under NFS reflects adaptive strategies that sustain metabolic function, such as maintaining nutrient uptake capacity in roots and providing N reserves in leaves capacity (Krapp 2015; Tegeder and Masclaux-Daubresse 2018; Peng et al. 2023a). The enhanced GS activity, peaking at night, likely contributes to the observed increased NH_4_^+^ levels in aboveground tissues and roots at 22:00, supporting N status during the dark period when photosynthesis is not active (Miflin and Habash 2002). Understanding these spatial patterns of N partitioning patterns and their underlying regulatory mechanisms, such as diurnal rhythms and coordinated enzyme regulation, is essential for guiding crop improvement strategies. Future studies will delve into the molecular pathways specifically modulating these spatial patterns, particularly focusing on the mechanisms underlying N remobilization between source and sink tissues under more complex, continuous N fluctuations to better reflect natural field conditions.

### Coordinated C–N metabolism under NFS enhances maize growth

Furthermore, building on our previous study on C metabolism in maize under NFS (Amoah and Kaiser 2025b), this study integrates those findings with new insight into N metabolism to enhance our understanding of maize growth response under NFS. Although we previously demonstrated that NFS promotes dynamic C allocation and sucrose partitioning, the results of this study highlight how these C adjustments are linked with N assimilation pathways. The superior growth observed under NFS may arise from coordinated C allocation strategies that support N metabolism. Specifically, enhanced sucrose transport to the roots strengthens root sink capacity by supporting root growth, energy supply, and N assimilation processes (Stitt and Krapp 1999; Lalonde et al. 2003). Consequently, the accumulation of N metabolites; NO_3_^−^, NH_4_^+^, and NO_2_^−^ feeds back to stimulate C metabolism through the regulation of SPS, SuSy and INVs activity. This bidirectional reinforcement between C and N metabolism highlights the observed increases in amino acid biosynthesis, protein accumulation, biomass production and photosynthetic efficiency under NFS. Furthermore, the diurnal stabilization of N pools may help maintain rhythmic C partitioning, ensuring sustained resource availability for growth and development. By linking the C partitioning mechanisms identified in our previous study with the N metabolic adjustments revealed here, our findings demonstrate that NFS coordinates carbon and nitrogen metabolism into an integrated physiological response that drives enhanced growth performance in maize. These results highlight NFS as a viable strategy to improve crop productivity through synergistic regulation of C and N metabolism pathways. Future studies will delve into the molecular and enzymatic pathways specifically modulating these spatial patterns, particularly focusing on the mechanisms underlying N remobilization between source and sink tissues under more complex, continuous N fluctuations to better reflect natural field conditions.

## Conclusion

This study demonstrates that NFS significantly enhances overall maize performance, including shoot and root development, biomass accumulation, and photosynthetic capacity, outperforming static single or mixed nitrogen treatments. NFS plants exhibited increased total N levels and increased accumulation of NO_3_^−^, NO_2_^−^, and NH_4_^+^ in both leaves and roots, with these concentrations sustained across diel cycles. Importantly, NFS improves NUE by leveraging the alternating supply of NH_4_^+^ and NO_3_^−^ to modulate a synergistic interaction. This dynamic regime optimizes N acquisition while mitigating the stress and toxicity typically associated with prolonged exposure to a single N form. It promotes nutrient homeostasis and induces adaptive metabolic adjustments not observed under static conditions. Mechanistically, NFS enhances NH_4_^+^ assimilation via the GS-GOGAT pathway, facilitating amino acid biosynthesis and efficient N incorporation. Elevated activities of NR, NiR, GS, and GOGAT, underscore the plant’s capacity to fine-tune its N metabolic machinery in response to fluctuating availability. Tissue-specific analyses further revealed that under NFS, roots act as dynamic N sink, while leaves retain elevated NO_3_^−^ levels overnight, serving as temporary storage pools for subsequent remobilization. These spatial patterns, coupled with diurnal shifts in N metabolites and enzyme activities, suggest that NFS modulates the amplitude and efficiency of circadian-regulated N processes, ultimately enhancing N utilization. Collectively, these findings highlight the significant advantages of dynamic NH_4_^+^–NO_3_^−^ interactions over static nitrogen regimes, offering a promising strategy to improve NUE and support sustainable agriculture by better mimicking natural N fluctuations. Future research will focus on validating these results through field trials across different maize varieties and exploring the effects of more complex, continuous N fluctuations to truly simulate natural environments, thereby maximizing their applicability and impact. Additionally, transcriptomic analyses will offer deeper insights into the molecular mechanisms underpinning NFS responses, enabling a more comprehensive understanding of how dynamic N supply shapes plant function at multiple biological levels.

## Supplementary Information

Below is the link to the electronic supplementary material.

Supplementary Material 1

## Data Availability

Data is contained in the manuscript.
