# Correction: Nitrogen form substitution identifies nitrogen use efficiency management pathways in maize

**DOI:** 10.1007/s12298-026-01783-7

**Published:** 2026-06-29

**Authors:** Joseph N. Amoah, Claudia Keitel, Brent N. Kaiser

**Affiliations:** School of Life and Environmental Sciences, The University of Sydney, 380 Werombi Road, Brownlow Hill, Camden, NSW 2570 Australia

**Correction to: Physiology and Molecular Biology of Plants (May 2026) 32(5):1011–1023**

10.1007/s12298-026-01753-z

 In this article, Figs. 1–4 were incorrectly numbered. Specifically, Fig. 1 should have been presented as Fig. 4, Fig. 2 as Fig. 1, Fig. 3 as Fig. 2, and Fig. 4 as Fig. 3. The corrected figure order is provided below.

**Fig. 1:**
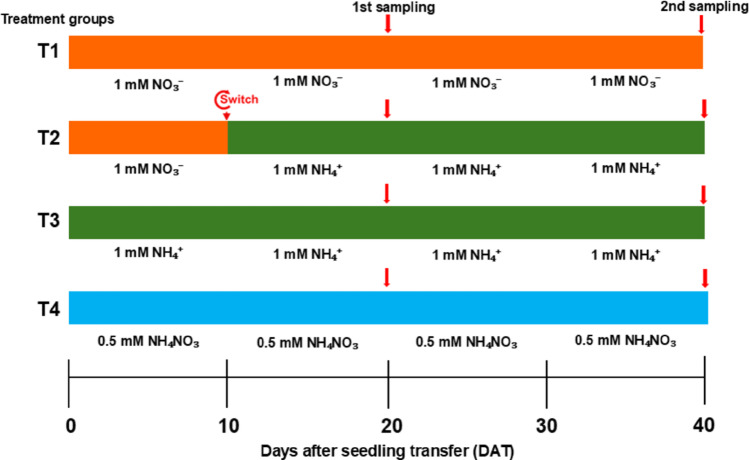
Schematic representation of the experimental setup for the maize inbred line TX-40 J. Maize seedlings were assigned to four nitrogen (N) treatment groups (T1 to T4): T1, 1 mM NO_3_^−^ (sole nitrate supply); T2, substitution of 1 mM NO_3_^−^ with 1 mM NH_4_^+^ at 10 days after transfer (DAT) (nitrogen form substitution, NFS); T3, 1 mM NH_4_^+^ (sole ammonium supply); and T4, 0.5 mM NH_4_NO_3_ (combined nitrate and ammonium supply). Plants were subsequently grown for an additional 30 days (up to 40 DAT), with shoot, root, and leaf samples collected at 20 DAT and 40 DAT. In the diagram, orange, green, and blue blocks represent 1 mM NO_3_^−^, 1 mM NH_4_^+^, and 0.5 mM NH_4_NO_3_, respectively. Red downward arrows indicate the nitrogen form switch (from 1 mM NO_3_^−^ to 1 mM NH_4_^+^), while black downward arrows denote tissue sampling time points

**Fig. 2:**
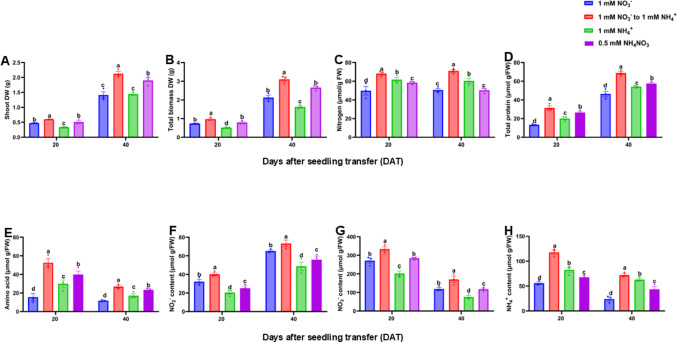
Shoot biomass (**A**), total biomass (**B**), leaf nitrogen content (**C**), leaf total protein content (**D**), leaf total amino acids (**E**), leaf nitrite (**F**), leaf nitrate (**G**), and leaf ammonium content (**H**) under different nitrogen forms. Data points represent the mean ± standard deviation (SD) of six independent biological replicates (*n* = 6). Different letters above the error bars indicate statistically significant differences at *P* ≤ 0.05. *DAT * days after seedling transfer;* FW* fresh weight;* DW* dry weight

**Fig. 3:**
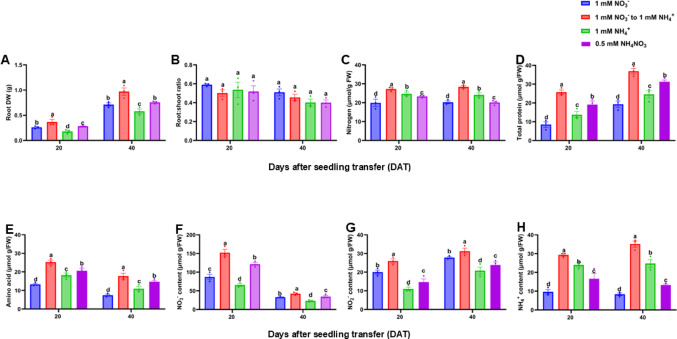
Root biomass (**A**), root: shoot biomass ratio (**B**), root nitrogen content (**C**), root total protein content (**D**), root total amino acids (**E**), root nitrate (**F**), root nitrite (**G**), and root ammonium content (**H**) under different nitrogen forms. Data points represent the mean ± standard deviation (SD) of six independent biological replicates (*n* = 6). Different letters above the error bars indicate statistically significant differences at *P* ≤ 0.05. *DAT* days after seedling transfer; *FW* fresh weight; *DW* dry weight

**Fig. 4:**
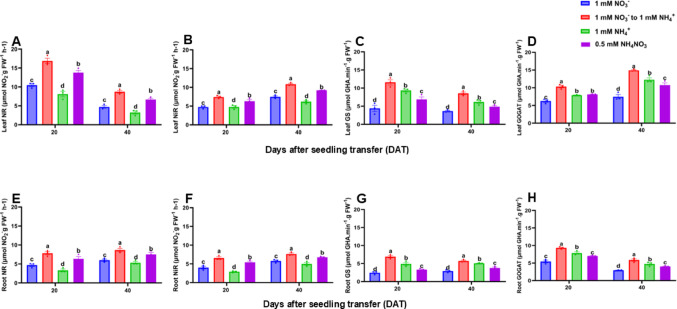
Nitrate reductase (**A**, **E**), nitrite reductase (**B**, **F**), glutamine synthetase (**C**, **G**), and glutamate synthase (**D**, **H**) activities in the leaves (**A**–**D**) and roots (**E**–**H**) of the maize inbred line TX-40 J. Data are presented as mean ± standard deviation (SD) from six independent biological replicates (*n* = 6). Different letters above the error bars indicate statistically significant differences at *P* ≤ 0.05. *DAT* days after seedling transfer; *FW* fresh weight

The original article has been corrected.

