# Supplementary material for: Nitrogen form substitution identifies nitrogen use efficiency management pathways in maize
Source: Physiol Mol Biol Plants. 2026 Apr 27;32(5):1011–23. doi: 10.1007/s12298-026-01753-z (PMC13216394; doi:10.1007/s12298-026-01753-z)
Supplement: Supplementary file 1 — Supplementary Material 1 [file 12298_2026_1753_MOESM1_ESM.docx]

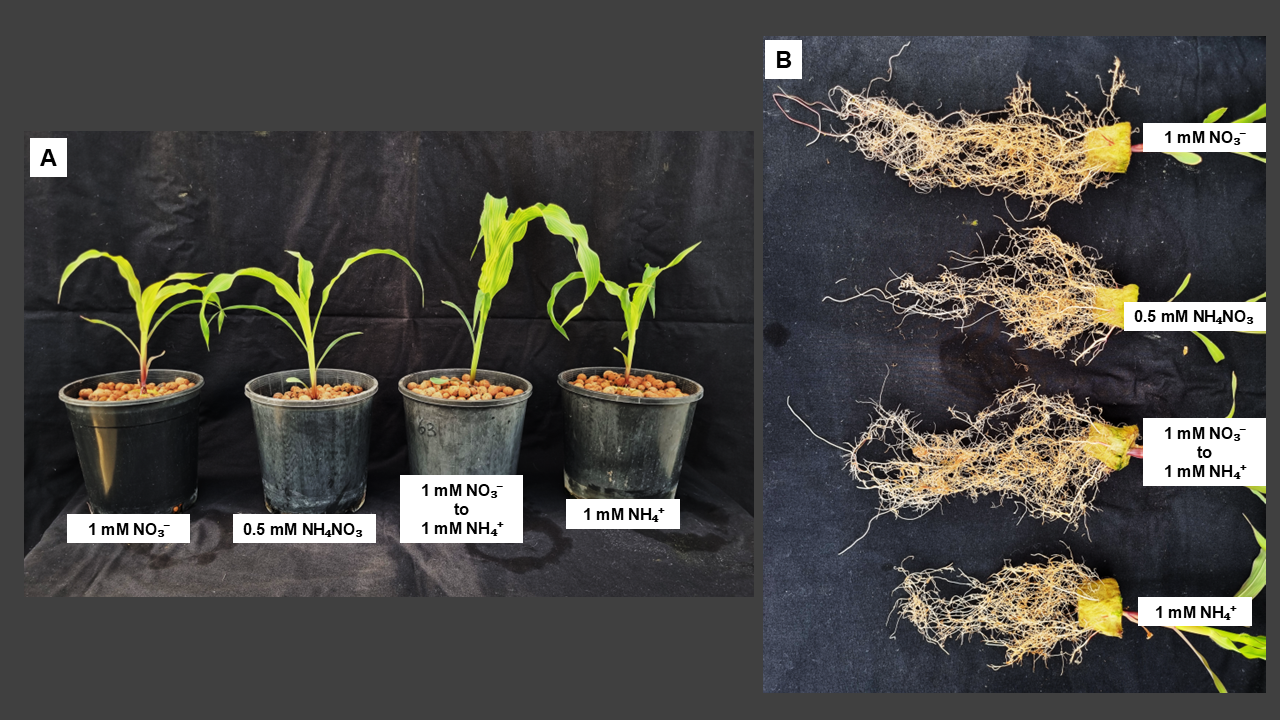


**Fig. S1:** Phenotypic response of the maize inbred line TX-40J to different nitrogen (N) forms. Shoot (A) and root (B) morphology at 20 days after nitrogen treatments (DAT).


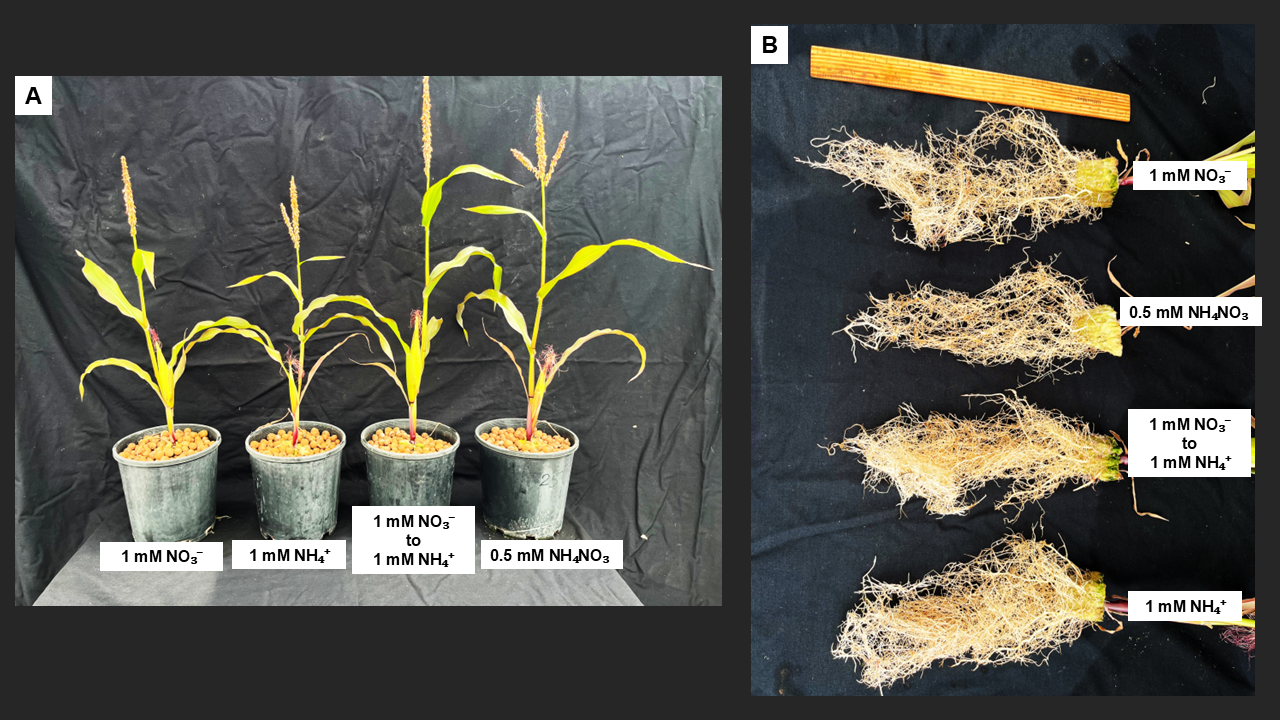


**Fig. S2:** Phenotypic response of the maize inbred line TX-40J to different nitrogen (N) forms. Shoot (A) and root (B) morphology at 40 days after nitrogen treatments (DAT).


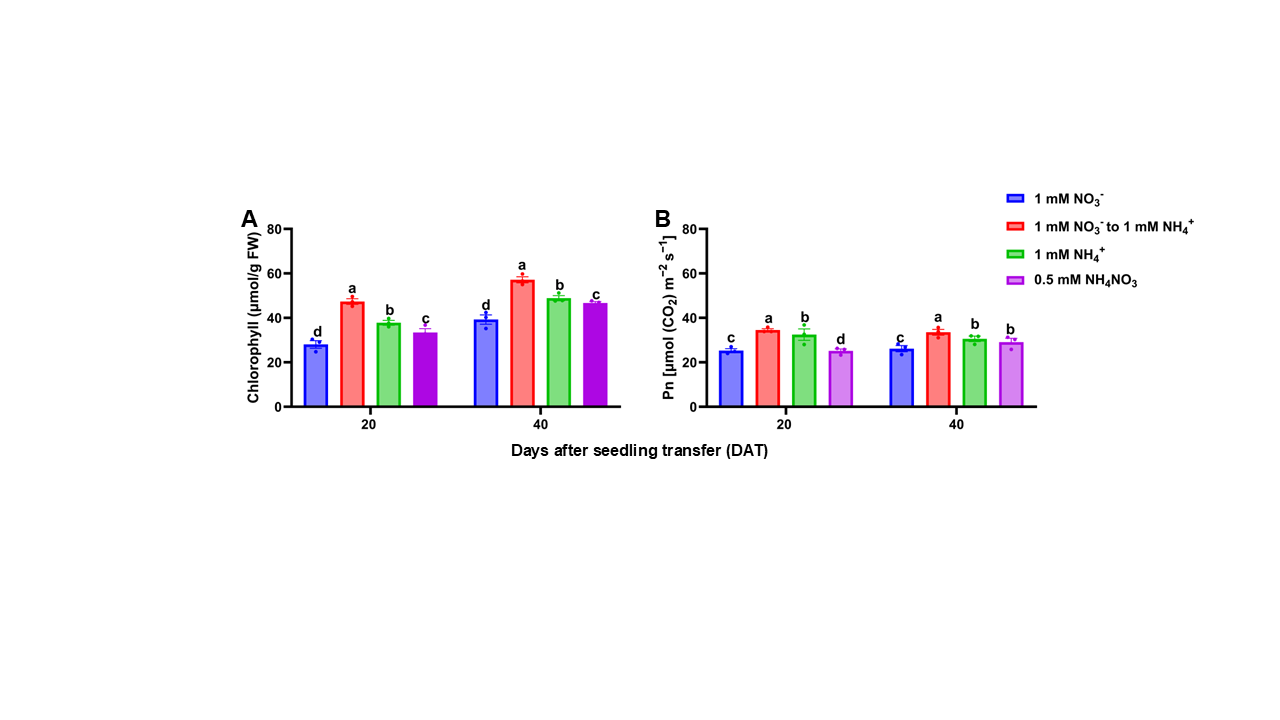


**Fig. S3:** Leaf chlorophyll content (A) and net photosynthetic rate (B under different nitrogen forms. Data points represent the mean ± standard deviation (SD) of six independent biological replicates (n = 6). Different letters above the error bars indicate statistically significant differences at *P* ≤ 0.05. Abbreviations: DAT – days after seedling transfer; FW – fresh weight.
